# Efficacy of membrane sweeping at term pregnancy to promote spontaneous labor: A comparative study

**DOI:** 10.12669/pjms.42.(ICON26).15699

**Published:** 2026-04

**Authors:** Moatter Asmat, Samina Mumtaz, Samiea Perveen

**Affiliations:** 1 Dr. Moatter Asmat, Department of Gynaecology and Obstetrics, Recep Tayyip Erdogan Hospital, Muzaffargarh 03422, Pakistan; 2 Dr. Samina Mumtaz, Department of Gynaecology and Obstetrics, Recep Tayyip Erdogan Hospital, Muzaffargarh 03422, Pakistan; 3 Dr. Samiea Perveen, Department of Gynaecology and Obstetrics, Recep Tayyip Erdogan Hospital, Muzaffargarh 03422, Pakistan

**Keywords:** Delivery, Membrane sweeping, Spontaneous labor, Term pregnancy

## Abstract

**Background & Objective:**

Membrane sweeping is done to promote spontaneous initiation of labor at term to avoid post-date pregnancy which is associated with perinatal morbidity and mortality. Post-date pregnancy occurs in 4–18% cases. This study will help in reducing rates of cesarean sections and post-term pregnancies and its associated complications. Our objective was to determine frequency of initiation of labor by membrane sweeping in low-risk pregnant women at term as compared to initiation of labor in pregnant women having expectant management till 40 weeks

**Methodology:**

A comparative study was done in outpatients visiting Obstetrics and Gynecology department in Recep Tayyip Erdogan Hospital, Muzaffargarh between June 1, 2024 to December 31, 2024. One hundred and eighteen uncomplicated pregnant patients, randomly allocated at 37 weeks into study and control groups, Group-A membrane sweeping (n=59), Group-B without membrane sweeping (n-59). In Group-A, membrane sweeping done on weekly antenatal visit starting at 37 week and control group received standard care with no membrane sweeping. Primary outcome measures are frequency of patients going into spontaneous labor and gestational age at labor onset.

**Results:**

Frequency of women going into spontaneous labor was significantly higher in membrane sweeping group compared to no membrane sweeping (91.5% vs. 71.2%, p-value=0.005). The mean gestational age at delivery was significantly lower in membrane sweeping group compared to no sweeping group (38.2±0.5 vs. 39.8±1.2 weeks, p<0.001).

**Conclusion:**

Sweeping of the membranes at term increases the chance of women for going into spontaneous labor before 41 weeks and reduces the incidence of post-date gestation.

## INTRODUCTION

Membrane sweeping is a mechanical method of inducing labor at term, which can be easily performed as an outpatient procedure to prevent post-date pregnancy. Post-date pregnancy, defined as a gestational period extending beyond 42 weeks, is associated with an increased risk of perinatal mortality and morbidity.^1^ According to the National Institute for Health and Care Excellence (NICE) guidelines, women should be informed that inducing labor at 41+0 weeks may reduce these risks.^2^ Membrane sweeping is an effective method of inducing labor in uncomplicated term pregnancies where induction is not urgently required. Although it is less effective than other methods, such as oxytocin, amniotomy, or prostaglandins, membrane sweeping can reduce the need for formal labor induction and promote spontaneous labor. During the procedure, the clinician separates the inferior pole of the membranes from the lower uterine segment, triggering the release of endogenous prostaglandins and oxytocin, which ripens the cervix and stimulates uterine contractions.^3^

Previous studies have demonstrated the efficacy of membrane sweeping in reducing the need for labor induction and promoting spontaneous labor. A study conducted in India reported a spontaneous labor rate of 90% in the membrane sweeping group, compared to 67.1% in the control group.^4^ Another study found 97.9% of women who underwent membrane sweeping had a spontaneous onset of labor before 41 weeks, compared to 46% in the control group.^5^

The most frequent cause of pharmacological induction of labor is post-term pregnancy, and membrane sweeping is an easy and affordable way to lessen this. The rate of caesarean sections rising as a result of unsuccessful labor induction, just as the rate of pharmacological induction of labor is rising.^6^

Given the increasing rates of pharmacological induction of labor and associated caesarean sections, this study aimed to investigate whether membrane sweeping, initiated at 37 weeks of gestation and performed weekly until 40 weeks, can reduce the need for IOL and associated caesarean section and promote spontaneous onset of labor.

## METHODOLOGY

Thi**s** comparative study was conducted in outpatients visiting Obstetrics and Gynaecology Department in Recep Tayyip Erdogan Hospital, Muzaffargarh between June 1, 2024 to December 31, 2024 to see efficacy of membrane sweeping on term patients for spontaneous onset of labor.

### Ethical statement:

The study conducted after getting ethical approval from Indus hospital and health network international review board, approval no. IHHN_IRB_2023_05_014 approval date May 30, 2024.

***Sample size:***^4^ Sampling strategy used was Non probability consecutive sampling. According to reference study, 60 cases and 85 in comparison group. Ratio of sample size unexposed/exposed is 1:1 Confidence level: 95%, Power of study: 80%, Percent of exposed with outcome: 90 %, Percent of unexposed with outcome: 67.1%. So, my sample size Fleiss with CC, total 118 sample size. Group-A, n= 59 Group-B, n= 59.

### Inclusion criteria:

- Maternal age 18-40 years
- Singleton low risk pregnancy, cephalic presentation, between 37-40 weeks of gestation. (assessed on antenatal scan)
- On per abdminal examination: fetal head engaged 2/5 palpable and on per vaginal examination: amniotic membranes intact.

### Exclusion criteria:

- History of previous two caesarean sections
- Multiple gestation (on antenatal scan)
- Placenta previa (on antenatal scan)
- Diabetes and hypertension.

### Data collection:

One hundred eighteen patients were enrolled in the study. After getting informed consent from eligible patients, all patients were divided into two groups i.e., A and B. Age and gestational age was calculated from last menstrual period and dating scan. In Group-A, membrane sweeping was performed starting at 37 completed weeks. If cervix was admitting one finger, then 360 degrees rotation of finger was done to separate membranes form lower uterine segment. If cervix was tightly closed and did not allow admitting finger then cervical stretching and messaging was done and patient was followed on weekly basis and sweeping was done weekly and AFI was assessed until delivery or till 40 weeks. In Group-B, no pelvic examination was done till 40 weeks or patients going into spontaneous labor and gestational age at time of delivery was recorded in both groups. All data was recorded in Performa.

All collected data was entered in SPSS 26 and was analyzed. Mean and SD was calculated for variables like age, gestational age at delivery. Frequency and percentages were calculated for categorical variables like parity, previous scar and onset of spontaneous labor between 37-40 weeks. Comparison of outcome between both groups was done by chi-square test. chi-square test was applied to determine effect of membrane sweeping on initiation of labor. Level of significance was defined as 0.05.

## RESULTS

Total numbers of patients recruited for study was 118, which included 59 cases and 59 controls. Characteristics of pregnant women at term are tabulated. (Table-I).

**Table-I:** Characteristics of pregnant women at term presenting to the labor room (N=118).

Characteristics	Overall (N=118)	Membrane Sweeping Done (n=59)	Membrane Sweeping Not Done (n=59)	p-value*
Age (years)	26.9 ± 4.8	26.5 ± 5.0	27.4 ± 4.5	0.292
***Parity***				0.170
Primipara	24 (20.3)	15 (62.5)	9 (37.5)	
Multipara	94 (79.7)	44 (46.8)	50 (53.2)	
***Previous Uterine Scar***				0.364
Yes	5 (4.2)	4 (80)	1 (20)	
No	113 (95.8)	55 (48.7)	58 (51.3)	
Gestational Age at delivery (week)	39.0 ± 1.2	38.2 ± 0.5	39.8 ± 1.2	< 0.001

* Independent sample t-test for quantitative comparison, chi-square test) for qualitative comparison.

Overall, 81.4% (n=96) women delivered before 41-weeks of gestation. Frequency of women delivering before 41-weeks of gestation was significantly higher in membrane sweeping group compared to no membrane sweeping (91.5% vs. 71.2%, p-value 0.005 (Table-II).

**Table-II:** Outcome of pregnant women at term presenting to the labor room (N=118)

Characteristics	Spontaneous labor before 41-weeks		p-value*
	Yes	No	
Overall (N=118)	96 (81.4)	22 (18.6)	--
Membrane Sweeping (Yes)	54 (91.5)	5 (8.5)	0.005
Membrane Sweeping (No)	42 (71.2)	17 (28.8)	

After stratification on parity status, there was no difference in frequency of delivery before 41-weeks in either of membrane sweeping or no sweeping group. However, in multiparous women the frequency of delivery before 41-weeks was significantly high in membrane sweeping compared to no membrane sweeping (93.2% vs. 68%, p-value 0.004) (Table-III).

**Table-III:** Effect of parity on distribution of outcomes between membrane sweeping and no sweeping groups (N=118).

Parity	Treatment Group	Spontaneous labor before 41-weeks		p-value*
		Yes	No	
Primiparous	Membrane Sweeping	13 (86.7)	2 (13.3)	1.00
	No Membrane Sweeping	8 (88.9)	1 (11.1)	
Multiparous	Membrane Sweeping	41 (93.2)	3 (6.8)	0.004
	No Membrane Sweeping	34 (68)	16 (30)	

* Fischer’s exact test.

## DISCUSSION

We observed in this study that frequency of women delivering before 41 weeks of gestation was significantly higher in membrane sweeping group compared to no membrane sweeping (91.5% vs. 71.2%, p=0.005). While the mean gestational age at delivery was significantly lower in membrane sweeping group compared to no sweeping group (38.2 ± 0.5 vs. 39.8 ± 1.2 weeks, p-value < 0.001).

Preventing post-term pregnancies with outpatient membrane sweeping at term is linked to shorter pregnancy durations. Studies in the literature suggest that prenatal cervical sweeping of the membranes can shorten the time it takes for labor to start on its own. A review of 22 studies (2,797 women) found that membrane sweeping, compared to no treatment, significantly reduced pregnancies continuing past 41 and 42 weeks, decreasing the need for formal labor induction (NNT=8), with similar risks for C-sections, but caused more discomfort, bleeding, and irregular contractions, though generally safe for low-risk term pregnancies.^7^ One study by de Miranda et.al reported that weekly sweeping puts women in a pre-labor state where irregular uterine contractions have a cervical ripening effect and improve the bishop score on admission.^8^

In a systemic review and meta-analysis study, Twice-weekly membrane sweeping was associated with increase in onset of spontaneous onset of Labor and decline in Labor induction in comparison with once-weekly group. Duration from sweeping to delivery was shorter among the twice-weekly group (p<0.001). Good Bishop score at admission was observed in the twice-weekly group (p=0.02).^9^

In a similar study researchers found that when membrane sweeping was used, there was a significant drop in the incidence of post-date pregnancy at 41 weeks, along with a lower induction rate (10% vs. 25%; P = 0.01) and a significant decrease in mean gestational age (39.5 ± 0.9 weeks versus 40.0 ± 1.2 weeks; P = 0.004).^10^ Their findings align with what we found.

A randomized controlled trial, concluded that sweeping the membranes is a safe and effective method for reducing post-dates pregnancy in low-risk populations. The findings indicated that this procedure does not increase the risk of adverse outcomes for either the mother or the newborn.^11^ In a study of 120 women from Pakistan, membrane sweeping significantly reduced the need for formal labor induction, with 106 women (88.3%) going into spontaneous labor, demonstrating its effectiveness in naturally starting labor.^12^

According to a recent study, twice-weekly group’s sweeping to the delivery interval (7.4 days) was considerably shorter than that of the once-weekly (8.8 days) and control groups (10.6 days). The twice-weekly group had significantly increased odds of spontaneous labor onset (HR 1.53, p =.029) than the control group (HR 0.65, p =.033).^13^

A meta-analysis showed that membrane sweeping is effective in inducing spontaneous labour and significantly reducing formal induction in post-term pregnancy. A review of seven studies with 2252 participants found that membrane sweeping helps start labour naturally (RR=1.205) and reduces the need for formal induction for pregnancies going past term (RR=0.523), with very strong statistical significance (p < .001 for both), indicating it’s beneficial for promoting spontaneous delivery and preventing prolonged pregnancy.^14^

In contrast to our findings, one study found no evidence of a substantial clinical impact on the length of pregnancy following membrane sweeping at 39 weeks of gestation. In the sweeping group, the mean time between sweeping (stripping) and vaginal examination till birth was 7.7 ± 6.9 and 7.1 ± 5.6 days, respectively (p = 0.61).^15^

### Limitations

It includes small sample size, non-blinded and single site data. The way forward is multi center randomized clinical control trail and larger number of pregnant females.

## CONCLUSION

Sweeping of the membranes at term increases the chance of women for going into spontaneous labor before 41 weeks and reduces the incidence of post-date gestation. While safer and less invasive than other labor induction techniques, sweeping the membranes improves an unfavorable cervix at term.

### Authors` Contribution

**MA:** Manuscript writing and responsible for the accuracy and integrity of the study.

**SM:** Literature search, did review and final approval of manuscript.

**SP:** Performed statistical analysis of data.

All authors have read and approved the final version.
